# Toxicological aspects of the use of phenolic compounds in disease prevention

**DOI:** 10.2478/v10102-011-0027-5

**Published:** 2011-12

**Authors:** Zuzana Kyselova

**Affiliations:** Institute of Experimental Pharmacology & Toxicology, Slovak Academy of Sciences, SK-84104 Bratislava, Slovakia

**Keywords:** phenolic compounds, pro-oxidant, estrogenicity, cytotoxicity, apoptosis

## Abstract

The consumption of a diet low in fat and enhanced by fruits and vegetables, especially rich in phenolic compounds, may reduce risks of many civilization diseases. The use of traditional medicines, mainly derived from plant sources, has become an attractive segment in the management of many lifestyle diseases. Concerning the application of dietary supplements (based on phenolic compounds) in common practice, the ongoing debate over possible adverse effects of certain nutrients and dosage levels is of great importance. Since dietary supplements are not classified as drugs, their potential toxicities and interactions have not been thoroughly evaluated. First, this review will introduce phenolic compounds as natural substances beneficial for human health. Second, the potential dual mode of action of flavonoids will be outlined. Third, potential deleterious impacts of phenolic compounds utilization will be discussed: pro-oxidant and estrogenic activities, cancerogenic potential, cytotoxic effects, apoptosis induction and flavonoid-drug interaction. Finally, future trends within the research field will be indicated.

## Introduction

Phenolic compounds are constituents of fruits, vegetables, nuts, plant-derived beverages – tea and wine, and traditional Eastern medicines such as Ginkgo biloba, as well as components present in a plethora of herbal dietary supplements. Humans have consumed flavonoids and other dietary phenolics since the arrival of human life on earth. Over 4 000 different naturally occurring phenolic compounds have been described (Middleton & Kandaswami, 1986) and the list is still growing.

Plants produce phenolic compounds as secondary metabolites involved in diverse processes, such as growth, lignification, pigmentation, pollination, and resistance against pathogens, predators, and environmental stresses (Duthie *et al.*, 2003; Fraga *et al.*, 2010).

Phenolic compounds have been known as plant pigments for over a century and belong to a vast group widely distributed in all foods of plant origin. In the normal North American diet they are unavoidably consumed daily with an estimated total consumption of 1 g/d (Formica & Regelson, 1995), which could be much higher if dietary supplements are also consumed.

**Figure 1:**
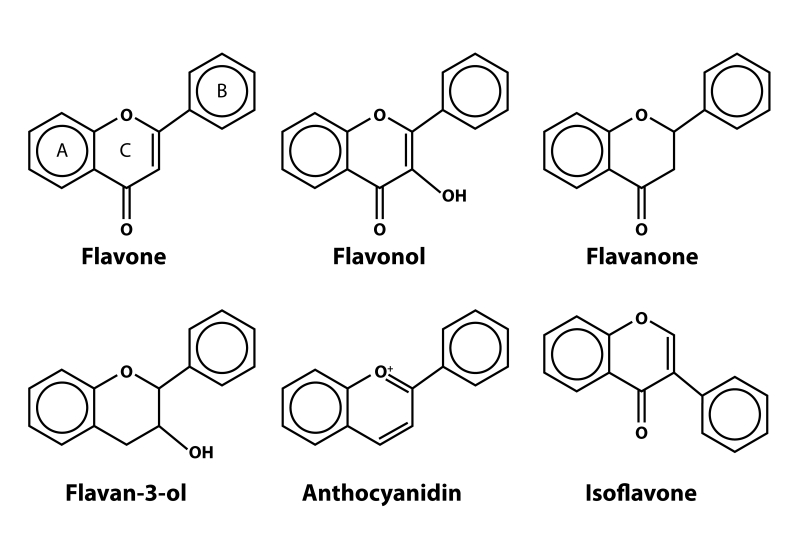
Various types of common flavonoids, basic structures of flavone, flavonol, flavanone, flavan-3-ol, anthocyanidin and isoflavone. At the structure of flavone marks for benzene rings: A, B, and C are indicated.

Chemically, phenolic compounds have one or more hydroxyl groups attached to a benzene ring. Edible plants contribute to the human diet more than 8 000 different phenolic compounds that can be categorized as flavonoids and non-flavonoid compounds. Flavonoids have a common C6–C3–C6 structure consisting of two aromatic rings (A and B) linked through a three-carbon chain, usually organized as an oxygenated heterocycle (ring C) (Figure 1). Flavonoids can be divided into several subfamilies according to the degree of oxidation of the oxygenated heterocycle as flavanols, flavanones, flavones, flavonols (essentially, flavan-3-ols), isoflavones, and anthocyanidins, the most relevant for human diets (Scalbert & Williamson, 2000). Starting from a basic chemical structure, plant biosynthetic pathways introduce different hydroxyl group patterns, methyl groups, and sugars (Jaganatah & Crozier, 2010). In certain cases, oligomerization and polymerization of the flavonoid units occur. Oligomers and polymers of flavonoids are called tannins and are classified into two groups, condensed tannins and hydrolyzable tannins. Condensed tannins (also known as proanthocyanidins or procyanidins) are oligomers of flavanols, and their chemical structures are defined not only by the kind of monomer, but also according to the kind of link among monomers. There are several oligomerization patterns and some plants present characteristic manners of oligomerization, *e.g.* in cocoa the monomeric units are linked through 4→8 carbon–carbon bonds forming mostly B-type dimers (Jaganatah & Crozier, 2010). Hydrolyzable tannins are polymers readily hydrolyzed by acids into their components: a central core constituted by a polyol (a sugar, generally D-glucose, or a flavonoid, as catechin) and a phenolic carboxylic acid esterifying partially or totally the core molecule. These tannins are classified according to the phenolic carboxylic acids present, which could be gallic acid (gallotannins) or ellagic acid (ellagitannins) (Jaganathan & Mandal, 2009).

Overall compelling data from *in vitro* and *in vivo* laboratory studies, epidemiological investigations, and human clinical trials indicate that phenolic compounds are beneficial for human health. A large number of studies have identified cellular targets that could be involved in the health promoting actions of dietary plant phenolic compounds. Epidemiological evidence indicates that diets rich in fruits and vegetables promote health and attenuate or delay the onset of many diseases (Knekt *et al.*, 2002; Ghosh & Konishi, 2007; Rhone & Basu, 2008; Patil *et al.*, 2009; Agte & Tarwadi, 2010; Cvorovic *et al.*, 2010; Crozier *et al.*, 2010; Ziberna *et al.*, 2010; Kalt *et al.*, 2010). The beneficial effects of fruits and vegetables have been largely ascribed to phenolic compounds, since ingestion of foods rich in these substances has been associated in humans and experimental animals with reduction in: i) dyslipidemia and atherosclerosis; ii) endothelial dysfunction and hypertension; iii) platelet activation and thrombosis; iv) inflammatory processes associated with induction and perpetuation of cardiovascular diseases (Fraga *et al.*, 2010).

Guarrera *et al.* (2007) found a flavonoid-rich diet to be likely to have beneficial health effects through modulating the expression of certain genes that have been clearly related to disease risk. Likewise, Halliwell (2007) summarized in his review the potential of phenolic compounds to protect against cardiovascular disease, supporting this conception by much convincing evidence from a number of pre-experimental models.

Anthocyanidins have been suggested as useful agents in disease prevention (Wang & Stoner, 2008; Cvorovic *et al.*, 2010). Recent studies showed their ability to trigger apoptosis in human leukemia cell lines through induction of oxidative stress (Hou *et al.*, 2003; Feng *et al.*, 2007) and their potential role in cancer therapy (Syed *et al.*, 2008).

With the increasing interest in alternative medicine, herbal products are ingested by at least 10% of the general population and 30–70% of individuals with specific disease states (Ni *et al.*, 2002; Galati & O′Brien, 2004). However, dietary supplements are not classified as drugs and do not require Food and Drug Administration (FDA) approval to be marketed. The potential toxicities and drug interactions have not been evaluated thoroughly, yet they should be considered. Thus, the main goal of this review will be to focus on different possible deleterious impacts of flavonoid utilization: pro-oxidant and estrogenic activities, cancerogenic potential, cytotoxic effects, apoptosis induction and flavonoid-drug interaction.

## Potential dual mode of action of phenolic compounds

Phenolic compounds might exert modulatory effects in cells independent of classical antioxidant capacity through selective actions at different components of a number of protein kinase and lipid kinase signaling cascades such as phosphoinositide 3-kinase (PI 3-kinase), Akt/PKB, tyrosine kinases, protein kinase C (PKC), and mitogen-activated protein kinases (MAPKs) (Kong *et al.*, 2000; Schroeter *et al.*, 2001; Spencer *et al.*, 2003a). Inhibitory or stimulatory actions at these pathways are likely to profoundly affect cellular function by altering the phosphorylation state of target molecules and/or by modulating gene expression. Although selective inhibitory actions at these kinase cascades may be beneficial in cancer, proliferative diseases, inflammation, and neurodegeneration, they could be detrimental during development, particularly in the immature nervous system, when protein and lipid kinase signaling regulates survival, synaptogenesis, and neurite outgrowth (Williams *et al.*, 2004). Thus flavonoid interactions with intracellular signaling pathways could have unpredictable outcomes, depending on the cell type, the disease studied, and the stimulus applied.

Antioxidant activity of phenolic compounds can be described directly, in terms of their *per se* activity as free radical scavengers, or indirectly as modulators of intracellular pro- and anti-oxidant enzymes (Schewe *et al.*, 2008). However, in recent years, both antioxidant and pro-oxidant activities were reported, depending upon certain experimental conditions (Halliwell, 2008; Ziberna *et al.*, 2010). Often these pro-oxidant effects involve interactions of phenolic compounds with transition metal ions, which can be ubiquitously found in biologic systems, or as universal contaminants of biologic reagents (Halliwell, 2009).

Kong *et al.* (2000) provided examples of many natural products eliciting diverse pharmacological effects. Studying two classes of potential chemopreventive compounds, phenolic compounds and isothiocyanates, they reviewed the potential utility of two signaling events, MAPKs and ICE/Ced-3 proteases (caspases) stimulated by these agents in mammalian cell lines. Studies with phenolic antioxidants (butylated hydroxyanisole – BHA, tert-buthyl hydrochinone – tBHQ) and natural products (either flavonoids epigallocatechin-3-gallate – EGCG and epicatechin 3-gallate – ECG or isothiocyanates phenethyl isothiocyanate **–** PEITC and sulforaphane) provided important insights into the signaling pathways induced by these compounds. At low concentrations, these chemicals activated the MAPKs (ERK2, JNK1, p38) leading to expression of genes provoking protective mechanisms (homeostasis response). However, to the contrary, on increasing their concentrations, these compounds additionally activated the caspase pathway, leading to apoptosis (potential cytotoxicity). Further increments to supra-pharmacological concentrations would inevitably lead to nonspecific necrotic cell death. This study revelaed the respective *in vivo* therapeutic window for the compounds tested and also extended knowledge about their efficacy and safety.

Another very good example is the flavonol quercetin – one of the most frequently researched flavonoids, with well described beneficial (Oyama *et al.*, 1999) and/or deleterious effects (Metodiewa *et al.*, 1999; Rzymowska *et al.*, 1999; Rong *et al.*, 2000; Spencer *et al.*, 2003b) on different cell types. Its exact mechanism of action remains however still unclear. Quercetin has been thoroughly investigated for its abilities to express antiproliferative effects (Kuo, 1996; Csokay *et al.*, 1997) and induce cell death predominantly by an apoptotic mechanism in cancer cell lines (Wei *et al.*, 1994; Wang *et al.*, 1999; Russo *et al.*, 1999; Williams *et al.*, 2004).

Based on the structural properties of quercetin, Metodiewa *et al.* (1999) investigated whether its catechol moiety may be the potential tool for the revealed toxicity. Their results were indicative of intracellular metabolic activation of quercetin to o-quinone, a process which can be partially associated with its observed concentration-dependent cytotoxic effect.

Similarly, Spencer *et al.* (2003a) observed a dual quercetin effect on intracellular signaling pathways such as the MAPKs cascade. They investigated whether the observed strong neurotoxic potential of quercetin in primary cortical neurons may occur *via* specific and sensitive interactions within neuronal mitogen-activated protein kinase and Akt/protein kinase B (PKB) signaling cascades, both implicated in neuronal apoptosis. High quercetin concentrations (30 µM) led to sustained loss of Akt phosphorylation and subsequent Akt cleavage by caspase-3, whereas at lower concentrations (<10 µM) the inhibition of Akt phosphorylation was transient and eventually returned to basal levels. Lower levels of quercetin also induced strong activation of the pro-survival transcription factor cyclic adenosine monophosphate (cAMP)-responsive element-binding protein, although this did not prevent neuronal damage. The glucuronide of quercetin was not toxic and did not evoke any alterations in neuronal signaling, probably reflecting its inability to enter neurons.

Recently, Angelone *et al.* (2010) analyzed whether two red wine phenolic compounds, quercetin and myricetin, affect mammalian basal myocardial and coronary function. Quercetin induced dual inotropic and lusitropic effects, positive at lower concentrations and negative at higher concentrations. Contrarily, myricetin elicited coronary dilation, without affecting contractility and relaxation. Simultaneous administration of the two phenolic compounds induced only vasodilation. Quercetinelicited positive inotropism and lusitropism depend on beta1/beta2-adrenergic receptors and are associated with increased intracellular cAMP, while the negative inotropism and lusitropism observed at higher concentrations were alpha-adrenergic-dependent.

## Possible deleterious impacts of utilizing phenolic compounds

### Pro-oxidant activities

The chemopreventive properties of phenolic compounds are generally believed to reflect their ability to scavenge endogenous reactive oxygen species (ROS). However, the pro-oxidant action of plant-derived phenolics rather than their antioxidant action may be an important mechanism for their anticancer and apoptosis-inducing properties, as ROS can mediate apoptotic deoxiribonucleic acid (DNA) fragmentation (Rahman *et al.*, 1990; Haidi *et al.*, 2000; Ahmad *et al.*, 2000; Galati & O′Brien, 2004).

Certain properties of dietary phenolic compounds, such as binding and cleavage of DNA and the generation of ROS in the presence of transition metal ions (Rahman *et al.*, 1990) are similar to those of known anticancer drugs. A putative mechanism has been proposed for the anticancer and apoptosis-inducing properties of plant-derived dietary phenolic compounds, which is a mechanism of DNA fragmentation that involves the mobilization of intracellular and extracellular copper (Haidi *et al.*, 2000). As an example, the reaction mechanism of DNA cleavage by resveratrol and Cu^2+^ was investigated by researchers of another laboratory, who found that resveratrol formed a complex with Cu^2+^, reducing it to Cu^+^, with the formation of one or more ‘‘oxidized species’’ of resveratrol (Ahmad *et al.*, 2000). The authors then demonstrated that the oxidized products of resveratrol were capable of reducing Cu^2+^ to Cu^+^. When calf thymus DNA was treated with increasing molar ratios of Cu^2+^ and resveratrol, the concomitant efficiency of DNA cleavage was increased (Haidi *et al.*, 2000).

Dietary phenolics have been shown to act as pro-oxidants in systems containing redox-active metals. In the presence of oxygen (O_2_), transition metals such as copper and iron catalyze the redox cycling of phenolics, leading to the formation of ROS and phenoxyl radicals that can damage DNA, lipids, and other biological molecules (Decker, 1999; Yamanaka *et al.*, 1999). Exposure of DNA to dihydrocaffeic acid in the presence of copper resulted in more DNA single- and double-strand breaks than exposure to caffeic acid, whereas chlorogenic acid caused only minimal damage, although these phenolics had similar structures and redox potential (Sakihama *et al.*, 2002). The green tea catechin EGCG was recently shown to induce hydrogen peroxide (H_2_O_2_) generation and cause subsequent oxidative damage to isolated and cellular DNA in the presence of transition metal ions (Furukawa *et al.*, 2003).

Galati and O′Brien (2004) showed that upon oxidation by peroxidase/H_2_O_2_ catalytic concentrations of flavonoids with a phenol B ring (*e.g.* apigenin, naringenin) formed phenoxyl radicals and generated ROS. Moreover, they found that phenol ring-containing dietary flavonoids and other phenolics also caused glutathione oxidation in isolated rat hepatocytes (Galati *et al.,* 2002). Consumption of large amounts of phenolic compounds in the form of a concentrated supplement may not be considered safe until their *in vivo* potential for oxidative stress is evaluated.

### Estrogenic activities

Overall, flavonoids are considered to be nonestrogenic or weakly estrogenic. However, some flavones and flavonols (apigenin, kaempferol, and naringenin) act through estrogen-receptor mediated mechanisms and have been shown to have antiestrogenic effects similar to those of isoflavones in breast cancer cell cultures (Miksicek, 1995; Ross and Kasum, 2002). Apigenin and kaempferol are the most active flavonoids and inhibit estrone reduction at a concentration of 0.12 µmol/L (Makela *et al.*, 1998). Some flavones were shown to inhibit the 17β-oxidation of testosterone and estradiol to the less active steroids androstenedione and estrone. In addition, flavonoids inhibit placental aromatase (Kellis & Vickery, 1984; Ibrahim & Abdul-Hajj, 1990).

Isoflavones are diphenolic compounds structurally or functionally similar to endogenous estrogens and they display agonistic and antagonistic interactions with estrogen receptors (Wang *et al.*, 2008). Their biological activity is partly ascribed to the structural similarities with the primary physiologically relevant estrogen – 17β-estradiol (E2). They bind to and activate intracellular estrogen receptors ERα and ERβ and, mimicking the effects of estrogen, are commonly referred to as phytoestrogens. In most systems, the relative binding affinities of genistein and daidzein are greater for ERβ than for ERα, while E2 binds to both receptors with approximately equal affinities (Messina *et al.,* 2006). The estrogenic activities of soy isoflavones are thought to play an important role by their health-enhancing properties in menopausal symptoms and in treating osteoporosis (Lockwood, 2008).

Phytoestrogens are phenolic nonsteroidal plant compounds with estrogen-like biological activity. Most flavonoids are nonestrogenic or weakly estrogenic; however, the isoflavones such as genistein, other flavonoids such as apigenin and kaempferol, and the polyphenolic stilbenes such as resveratrol act through estrogen receptor-mediated mechanisms and also have antiestrogenic effects (Cos *et al.*, 2003). The use of phytoestrogens to protect against hormone dependent cancers or as a ‘‘natural’’ alternative to hormone replacement therapy remains controversial. There is a paucity of data on endocrine effects of soy phytochemicals such as genistein and daidzein during infancy, the most sensitive period of life for the induction of toxicity. The safety of isoflavones in infant formulas has been questioned recently owing to reports of possible hormonal effects (Miniello *et al.*, 2003). Closer studies are necessary both in experimental animals and human populations exposed to phytoestrogen containing products, and particularly to soy-based infant formulas, as the estrogenic activity of genistein at high doses has been shown to be associated with decreased fertility and increased sexual dysfunction in experimental animals (You *et al.*, 2002). There is also an increased risk for breast and reproductive tract cancers in humans. Estrogenic activity in soybean-related foodstuffs is mostly due to genistein, yet 8-prenylnaringenin, the active principle in beer, is 10 times more active than genistein (Takamura- Enya *et al.*, 2003).

Since overexposure to estrogen is a major contributing factor in the development of breast cancer, the relationship between soyfoods and breast cancer has become controversial. Concern has been raised that soy-derived isoflavones, which exhibit estrogen-like properties under certain experimental conditions, may stimulate the growth of existing estrogen sensitive tumors. This concern is substantiated by evidence showing that isoflavones bind and transactivate estrogen receptors and elicit estrogenic effects in rodent reproductive tissues. Limited human data directly address the tumor promoting effects of isoflavonoids (Messina *et al.*, 2006). Due to its estrogenic properties genistein at low physiologically relevant levels may stimulate estrogen receptor positive tumors, while at higher levels, anticancer actions of isoflavonoids may predominate (Duffy *et al.*, 2007).

Estrogen-like effects have raised concern regarding soy/isoflavone consumption, particularly in postmenopausal women at high risk of breast cancer. Currently, there is little evidence to suggest that any potential weak estrogenic effects of dietary isoflavones would have a clinically relevant impact on breast tissue in healthy women or in breast cancer survivors (Messina and Wood, 2008).

However, recent epidemiologic evidence showed that higher soy intake in Asian women was associated with a nearly one-third reduction in breast cancer risk and that Japanese breast cancer patients, in comparison to Western women, exhibited better survival rates even after the controll stage (Miadokova, 2009).

The developing fetus is partucularly sensitive to perturbation with estrogenic chemicals. The carcinogenic effect of prenatal exposure to diethylstilbestrol (DES) is a classic example: exposure to exogenous estrogens can induce both structural and functional changes in the developing reproductive tract of males. Men exposed to diethylstilbestrol (DES) *in utero*, for example, experienced DES-induced lesions ranging from relatively minor structural alterations to more severe changes such as testicular hypoplasia (Gill, 1988). It is possible that other exogenous estrogens, including flavonoids, may alter reproductive tract development in males.

Since phytoestrogen use in nutritional and pharmaceutical applications for infants and children is increasing, Newbold *et al.* (2001) investigated the carcinogenic potential of genistein, a naturally occurring plant estrogen in soy. In an experimental animal model they found a high incidence of uterine adenocarcinoma after neonatal DES exposure. Outbred female CD-1 mice were treated on postnatal days 1–5 with equivalent estrogenic doses of DES (0.001 mg/kg/day) or genistein (50 mg/kg/day). At 18 months, the incidence of uterine adenocarcinoma was 35% for genistein and 31% for DES. These data clearly indicate that genistein is carcinogenic if exposure occurs during critical periods of differentiation.

Thus, the use of soy-based infant formulas in the absence of medical necessity and the marketing of soy products designed to appeal to children should be closely examined. Given the increasing use and marketing of soy formulas, soy products, and phytoestrogen containing dietary supplements, further studies of the potential detrimental effects are needed.

### Cancerogenic potential

Flavonoids are phytochemicals that belong to popular chemopreventive compounds exerting a great variety of beneficial effects on human health, *e.g.* antiallergic, antiinflammatory, antiviral, antiproliferative, anticarcinogenic (Birt *et al.*, 2001). Their biological activities arise mainly from their antioxidant properties and abilities to modulate several enzymes or cell receptors (Klejdus *et al.*, 2005). Although flavonoids are often considered to be safe because of their “plant origin”, ingestion of flavonoids should be taken with caution. Some flavonoids show the ability of direct interaction with DNA and/or enhance carcinogenic activation into DNA modifying agents (Hodek *et al.*, 2006). Enhanced expression of cytochrome P450 (CYP) in colon tissue might be responsible for increasing incidence of colorectal carcinoma in humans (Hodek *et al.*, 2006). Some flavonoids have mutagenic (*e.g.* quercetin) and/or pro-oxidant effects and they may interfere with essential biochemical pathways (Rietjens *et al.*, 2005).

In spite of a long history of use, botanical or herbbased preparations may contain individual ingredients known to be toxic and even genotoxic and carcinogenic. The mutagenic properties of the flavonoid quercetin were demonstrated in a variety of bacterial and mammalian mutagenicity tests, and were related to its quinone/quinone methide chemistry (Brown, 1980). For quercetin a metabolic pathway for activation into DNA-reactive species may include enzymatic and/or chemical oxidation of quercetin to quercetin *ortho*-quinone, followed by isomerization of the *ortho*-quinone to quinone methides. These quinine methides are suggested to be the active alkylating DNA-reactive intermediates. Recent results showed formation of quercetin DNA adducts in exposed cells *in vitro* (Walle *et al.*, 2003).

Quercetin at higher doses was able to inhibit cell proliferation (van der Woude *et al.*, 2003). Evidence of its carcinogenic activity was found in male F344/N rats exposed to quercetin (Dunnick *et al.*, 1992). Moreover, Pamukcu *et al.* (1980) reported bladder tumors in rats exposed to quercetin. However, in several other animal studies no tumor initiating activity was reported (Morino *et al.*, 1982; Hirose *et al.*, 1983; Ito *et al.*, 1989). The relevance for the human *in vivo* situation as well as the mechanism behind the possible quercetin-mediated carcinogenic effect remains a matter of debate.

One of the questions that remains to be answered is why these genotoxic characteristics of quercetin obtained *in vitro*, and even carcinogenicity data obtained *in vivo* in animal studies using high doses of pure compounds, do not necessarily lead to carcinogenic risks.

It should be also noted that animal carcinogenicity experiments are conducted with pure compounds, whereas human dietary exposure generally occurs in a complex food matrix containing other (herbal) ingredients. In a complex food matrix, interactions occur that can affect the bioavailability of food components (Schiller *et al.*, 2003; Crozier *et al.*, 2010; Rietjens *et al.*, 2010). For example, a slow or incomplete release from the matrix could result in a reduced bioavailability as compared to bioavailability when dosed as a pure compound by oral gavage. In addition to the effect of the food matrix on the bioavailability, interactions with other herbal ingredients might occur at the level of metabolic activation and/or detoxification (Rietjens *et al.*, 2010).

The flavonoid genistein was one of the most active compounds tested that stimulated enzyme-mediated DNA cleavage approximately tenfold without requiring redox cycling for activity (Ferguson & Philpott, 2008). Flavonoids of this type have been suggested as etiological agents in specific types of childhood leukemia that is characterized by a distinctive chromosomal translocation (Ross *et al.*, 1994). Although epidemiology has provided some evidence for this hypothesis, it is difficult to be certain of the contribution of one component in a complex mixture. However, the recent finding (van Waalwijk van Doorn-Khosrovani *et al.*, 2007) that kaempferol, genistein, and quercetin induce myeloid/lymphoid or mixed-lineage leukemia translocations in primary human stem cells with characteristics of the translocations in this leukemia is entirely consistent with this hypothesis. Although the topoisomerase II inhibitory properties associated with quercetin might imply that it is an effective mutagen and potential dietary hazard, other studies point to antimutagenic effects. For example, Steiner *et al.* (2007) showed that preincubation of MCF-10A cells with genistein reduced DNA damage caused by two potential dietary mutagens – 4-hydroxy-2-nonenal (150 µM) and benzo(a)pyrene-7,8-dihydrodiol-9,10-epoxide (50 µM). The mechanism appeared to proceed through upregulation of glutathione-S-transferases, and the authors extrapolated these results to suggest the potential of this mechanism in preventing genotoxic injury in the etiology of breast cancer.

Induction of CYP by flavonoids might also significantly affect the plasma concentrations of pharmaceutical drugs, resulting in a loss of therapeutic effect or an overdose. CYP induction could also increase the metabolic activation of carcinogens. Certain flavonoids, like some other xenobiotics including 2,3,7,8-tetrachlorodibenzo-p-dioxin, induce CYPs *via* binding to aryl hydrocarbon receptor (AhR) (Kohn *et al.*, 2001). This mechanism is associated with an elevation in the activities of the CYP1 family enzymes that are responsible for the activation of carcinogens, such as meat-derived heterocyclic aromatic amines, benzo[a]pyrene, aflatoxin B1, and 7,12-dimethylbenz[a]anthracene (Omiecinski *et al.*, 1999). Many flavonoids are AhR ligands because the binding affinities for AhR seem to be largely dependent on structural constraints, including planar aromatic compounds with few bulky substituent groups (Waller and McKinney, 1995). Flavonoids have often been shown to act as AhR agonists and to induce CYP1A1 and CYP1A2 activities. Galangin, quercetin, diosmin, and diosmetin are examples of flavonoids that increase transcription of the CYP1A1 gene, whereas others, like flavone, tangeretin, and synthetic h-naphthoflavone, are flavonoids that induce CYP1A1/2 and to some extent CYP2B1/2 (Hodek *et al.*, 2002). Flavanone is a specific inducer of CYP2B1/2 (Canivenc-Lavier *et al.*, 1996). Aromatic amine carcinogens are metabolically activated by CYP1A2, whereas polycyclic aromatic hydrocarbon carcinogens are metabolically activated by CYP1A1 (Galati and O′Brien, 2004).

### Cytotoxicity

The cellular effects of flavonoids will ultimately depend on the extent to which they associate with cells, either by interactions at the membrane or by uptake into the cytosol. Information regarding uptake of flavonoids and their metabolites from the circulation into various cell types and their further assumed modification by cell interactions has become increasingly important as attention focuses on the new concept of flavonoids as potential modulators of intracellular signaling cascades vital to cellular function (Williams *et al.*, 2004).

Resveratrol and the citrus flavanones hesperetin and naringenin were reported to have inhibitory activity at a number of protein kinases (So *et al.*, 1996; Huang *et al.*, 1999; Fischer *et al.*, 2000). This inhibition is mediated *via* binding of the phenolic compounds to the ATP binding site, presumably causing three-dimensional structural changes in the kinase leading to its inactivity. Flavonoids may also interact with mitochondria, interfere with pathways of intermediary metabolism, and/or downregulate the expression of adhesion molecules (Panes *et al.*, 1996; Soriani *et al.*, 1998).

Cvorovic *et al.* (2010) recently observed a dual role for anthocyanidins, depending on cell type. In cells with low basal metabolic rates such as tumor cells with low proliferation rate and low malignant potency (*e.g.* Caco-2 cells), they behave as free radical scavengers and protect them from oxidative stress. On the other hand, in malignant cell, with active metabolism, high intrinsic ROS production, and drug-resistance (such as LoVo/ADR cells), delphinidin and cyanidin acted as pro-oxidants, putatively both by reduced capacity to scavenge reactive oxygen species and by depletion of the glutathione pool. Indeed, they found that delphinidin and cyanidin were cytotoxic in the metastatic human colorectal cancer cell lines LoVo and LoVo/ADR, where they inactivated the glutathione antioxidant system and promoted oxidative stress. Anthocyanidins were studied because in the colon they can be released from dietary anthocyanins by the action of glycosidases of the colonic microflora (Talavera *et al.*, 2005), though they are less stable than their glycosylated precursors at neutral pH, yielding phenolic aldehydes and phenolic acids (Woodward *et al.*, 2009), apparently mimicking *in vivo* conditions.

Similarly, Ziberna *et al.* (2010) described anthocyanins as exerting cardioprotective activities in low concentrations and cardiotoxic activities in high concentrations. They were first to report that high concentrations of anthocyanins exerted deleterious effects in ischemic-reperfusion injury on the isolated heart. They observed that bilberry anthocyanins induced a dual inotropism: positive at lower concentrations and negative at higher concentrations. This effect is in agreement with a similar study done with quercetin (Angelone *et al.*, 2010).

### Apoptosis induction

Another mechanism proposed for the anticancer and tumor cell apoptosis-inducing properties of flavonoids is that their pro-oxidant phenoxyl radicals cause mitochondrial toxicity by collapsing the mitochondrial membrane potential. Apoptosis (programed cell death) is required to maintain a balance between cell proliferation and cell loss. Misregulation of this balance can lead to malignant transformation, whereas induction of apoptosis suppresses the development of cancer (Tang & Porter, 1996; Bhat and Pezzuto, 2002). Various diet-derived compounds, *e.g.*, resveratrol, have been shown to induce apoptosis in malignant cells and provide a promising natural strategy to prevent cancer (Katdare *et al.*, 1999; Surh *et al.*, 1999).

It has been proposed that opening of the mitochondrial permeability transition pore (PTP) and/or mitochondrial membrane permeabilization (MMP) signals cell death by releasing apoptogenic factors, *e.g.*, cytochrome c, from the mitochondria. Dissipation of the mitochondrial membrane potential (Dcm) is an early event and the release of cytochrome c was shown to occur in a variety of models relatively early during cell death (Goldstein *et al.,* 2000). Cells with collapsed Dcm are irreversibly committed to undergo death, even when the apoptosis-inducing trigger is withdrawn, and this marks the point of no return in the cell death process (Kroemer, 2003). Curcumin, a dietary phenolic compound, also opened the PTP and induced mitochondrial swelling, calcium release, respiration impairment and collapsed the mitochondrial membrane potential (Kim *et al.*, 2003). Baicalin is a major component of the herbal medicine Sho-saiko-to, commonly used for treating chronic hepatitis in Japan and China. Baicalin induced apoptosis of Jurkat cells, a leukemia-derived T cell line, which was accompanied by intracellular ROS generation, mitochondrial cytochrome c release, and disruption of Dcm before activation of caspase 3. This suggests that baicalin acts as a pro-oxidant and induces mitochondrial-mediated apoptosis (Ueda *et al.*, 2001). Nordihydroguaiaretic acid (NDGA) found in chaparral, herbal medicine, is also a mitochondrial toxin (Biswal *et al.*, 2000). Furthermore, the isoflavone analog rotenone is a classical complex I inhibitor of the mitochondrial respiratory chain (Degli Esposti, 1998) and its analogs have been shown to be effective anticancer agents (Fang & Casida, 1998).

Epidemiological studies suggest that consumption of green tea is associated with a lower risk of several types of cancers, including stomach, esophagus, and lung (Chung *et al.*, 2001). The growth suppressive effect of green tea flavonoids and gallic acids is at least partly due to the induction of apoptosis (Paschka *et al.*, 1998).

Epigallocatechin was also shown to have antiproliferative and anticarcinogenic activities: mitochondrial depolarization and ROS formation were suggested to be early processes leading to EGCG-induced apoptosis of human prostate and lung carcinoma cells (Chung *et al.*, 2001; Yang *et al.*, 1998).

Flavopiridol is widely used in traditional medicine and is a novel semisynthetic flavone analog of rohitukine, a leading anticancer compound derived from an Indian tree. Flavopiridol caused apoptosis in human chronic lymphocytic leukemia cells, and the cytotoxic mechanism involved caspase 3 activation (Byrd *et al.*, 1998).

Wenzel *et al.* (2000) evaluated how the core structure of the flavones, 2-phenyl-4H-1-benzopyran-4-one, affected proliferation, differentiation, and apoptosis in a human colon cancer cell line. In particular, they evaluated the effect of the flavone on the expression of cell-cycle and apoptosis-related genes in the cell line. They reported dramatic changes in mRNA levels of specific genes including cyclo-oxygenase-2, nuclear transcription factor kappaB, and bcl-X. Further, there was high selectivity for apoptosis in the transformed cells. The authors concluded that flavones could be a new chemopreventive agent.

In addition to effects on mRNA levels of genes important in cell cycle control and apoptosis, flavonoids have been investigated with respect to their interaction with enzymes associated with DNA topology. DNA topoisomerase II is an enzyme that catalyzes the double-strand breakage and rejoining of DNA; it is pivotal for several cell functions (Gerwirtz, 1991). Several flavonoids, including genistein, can inhibit DNA topoisomerase II activity by stabilizing the cleavage complex, thereby facilitating apopotosis (Strick *et al.*, 2000). Importantly, however, specific flavonoids have been shown to cause single- and double-strand DNA breaks in cultured human cells (Strick *et al.*, 2000), suggesting that not all cells undergo apoptosis in the presence of specific flavonoids. This observation is extremely important in understanding the potential detrimental effects that may be associated with DNA topoisomerase II inhibition (Ross, 1998; Ross and Kasum, 2002).

### Interactions of phenolic compounds with drugs

Plants have long been used to treat some of the most common diseases in humans, but the effects of herbal medicinal products have not always been adequately investigated by pharmacological studies. For this reason the European Medicines Agency (EMA) is promoting European Community monographs on herbal medicinal products in order to assess their clinical safety and efficacy (Schilter *et al.*, 2003; EMEA Commitee on HMPC, 2006; Colalto, 2010). One safety threatening aspect is the risk of adverse effects due to pharmacological interactions between herbal medicinal products and conventional therapies (Brazier *et al.*, 2003). These are often underestimated for two main reasons: consumers generally consider herbal medicinal products “safe” because of their natural origin, and as self-care products they are often taken without consulting a physician (Rietjens *et al.*, 2008).

The efficacy of drug therapy depends on many factors related to a drug's pharmacokinetic and pharmacodynamic properties, which can be modified by differences in genetic polymorphisms, age, gender, circadian rhythms, intestinal bacteria, pathophysiological conditions, pharmaceutical dosage form and xenobiotics (Terada & Inui, 2007; Franconi *et al.*, 2007; Baraldo, 2008; Crozier *et al.*, 2010; Colalto, 2010). One particular case is the co-administration of traditional drugs and herbal medicinal products (*i.e.* dietary supplements containing medicinal herbs or the herbal medicines traditionally used in phytotherapy for treating or preventing diseases), which may cause unexpected interactions (Izzo & Ernst, 2001).

Inhibition of CYPs, which are necessary for carcinogen activation, is a beneficial chemopreventive property of various flavonoids, but it may be a potential toxic property in flavonoid-drug interactions (Galati & O′Brien, 2004). Inhibition of CYP activities by flavonoids has been extensively studied because of their potential use as agents blocking the initiation stage of carcinogenesis (Doostdar *et al.*, 2000). Flavonoids can either inhibit or induce human CYPs depending upon their structure, concentration, or experimental conditions. Thus *e.g.* α-naphthoflavone is an inhibitor of human CYP1A1 and 1A2 (Tassaneeyakul *et al.*, 1993) but an inducer of CYP3A4 (Guengerich *et al.*, 1994).

The simultaneous administration of flavonoids and clinically used drugs may cause flavonoid-drug interactions by modulating the pharmacokinetics of certain drugs, which results in an increase in their toxicity or a decline in their therapeutic effect, depending on the structure (Tang & Steams, 2001). Naringenin, a major flavanone present in grapefruit juice, exerts an inhibitory effect on intestinal CYP3A4 within 30 min and impairs the human metabolism of certain drugs such as those belonging to the class of calcium channel blockers (*e.g.*, felodipine, nitrendipine, nisoldipine, verapamil) when co-administered with grapefruit juice (Fuhr, 1998). Indiscriminate use of herbal products can also alter the pharmacokinetics of certain drugs and result in increased drug toxicity. This issue is particularly important in assessing the safety of concentrated flavonoid food supplements or herbal products particularly if their plasma concentrations stay high after ingestion (Hodek *et al.*, 2002; Galati & O′Brien, 2004). Inhibition of CYPs by flavonoids may also be a concern when non-therapeutic agents are consumed. By adding polar groups to these xenobiotics, CYPs play a role in enhancing their elimination. Although beneficial as a chemopreventive property, inhibition of CYPs by flavonoids may inhibit the metabolism and elimination of these nondrug compounds, increase their accumulation *in vivo*, and cause toxicity.

Phase II metabolism is generally regarded as a detoxification pathway and inhibition of these enzymes can lead to increased toxicity of a xenobiotic. Indeed, inhibition of glucuronosyltransferases (UGTs) by flavonoids, when taken concomitantly with a drug, can cause an overdose of the drug. Flavonoids have been shown to be substrates for UGTs and therefore, when taken in combination with certain drugs, they may inhibit their glucuronidation as a result of competitive inhibition (Galati & O′Brien, 2004).

## Natural substances beneficial for human health – future trends

In general, the consumption of a diet low in fat and enhanced by fruits and vegetables, especially rich in phenolic compounds, may reduce risks of many civilization diseases. Nowadays, informed patients increasingly demand substitution of prescription drugs by safer, often less expensive natural alternatives. Thus, the use of traditional medicines, mainly derived from plant sources, has become an attractive segment in the management of many lifestyle diseases.

Flavonoids may possess specific properties that can be beneficial for human health. However, as discussed, the experimental and *in vitro* data have produced conflicting results. The data from epidemiological studies regarding flavonoids in human health are far from convincing. This is especially disconcerting when specific flavonoids, such as genistein and quercetin are marketed as nutritional supplements, in which the concentrations in one dose could by far exceed the dose received from a daily vegetarian diet. The results of recent clinical studies should reinforce the need to proceed with caution in using flavonoid supplements. Whereas more studies at all levels are needed, to characterize both the potential health benefits of individual flavonoids and their potential harmful attributes, it is very possible that the sum of the parts (*e.g.* total fruit and vegetable intake) is more important in providing a health benefit to humans than one plant constituent.

However, concerning the application of flavonoid-based dietary supplements into common practice, the ongoing debate over possible adverse effects of certain nutrients and dosage levels is of utmost importance. Nutritionists caution that large doses of virtually any vitamin or mineral may affect the body's ability to absorb other nutrients and can be associated with some health risks. Thus toxicological aspects of unusually high doses for natural dietary supplements have to be assessed and the implication for public health considered. Future strategies looking into the synergistic effect of bioactive phytochemical components, specifically within the whole food matrix, fruits and/or vegetables including, would be desirable. In the meantime, incorporating 3–5 daily servings of either berry fruits or green leaf vegetables rich in flavonoids within the diet may be regarded as a proper choice for promoting health in the nowadays challenged organism.
